# Smoking and Age-Related Macular Degeneration: Review and Update

**DOI:** 10.1155/2013/895147

**Published:** 2013-12-04

**Authors:** Sara Velilla, José Javier García-Medina, Alfredo García-Layana, Rosa Dolz-Marco, Sheila Pons-Vázquez, M. Dolores Pinazo-Durán, Francisco Gómez-Ulla, J. Fernando Arévalo, Manuel Díaz-Llopis, Roberto Gallego-Pinazo

**Affiliations:** ^1^Department of Ophthalmology, San Pedro Hospital, Carretera de Piqueras 98, 26006 Logroño, Spain; ^2^RETICS Oftared, Instituto de Salud Carlos III, 28029 Madrid, Spain; ^3^Department of Ophthalmology, Reina Sofia General University Hospital, Avenida Intendente Jorge Palacios 1, 30003 Murcia, Spain; ^4^Department of Ophthalmology and Optometry, Faculty of Medicine, University of Murcia, 301000 Murcia, Spain; ^5^Ophthalmic Research Unit “Santiago Grisolía,” Department of Surgery/Ophthalmology, Faculty of Medicine and Odontology, University of Valencia, Avenida Blasco Ibanez 15, 46010 Valencia, Spain; ^6^Department of Ophthalmology, University Clinic of Navarra, Avenida de Pio XII 36, 31008 Pamplona, Spain; ^7^Department of Ophthalmology, University and Polytechnic Hospital La Fe, Carretera Bulevar Sur, 46026 Valencia, Spain; ^8^Faculty of Medicine and Odontology, University of Valencia, Avenida Blasco Ibanez 15, 46010 Valencia, Spain; ^9^Gómez-Ulla Institute of Ophthalmology, Calla Maruxa Mallo 3, 15706 Santiago de Compostela, Spain; ^10^University of Santiago de Compostela, Carretera San Francisco, 15705 Santiago de Compostela, Spain; ^11^Retina Division, Wilmer Eye Institute, Johns Hopkins University School of Medicine, Baltimore, MD 21205, USA; ^12^Vitreoretinal Division, King Khaled Eye Specialist Hospital, Riyhad 12329, Saudi Arabia

## Abstract

Age-related macular degeneration (AMD) is one of the main socioeconomical health issues worldwide. AMD has a multifactorial etiology with a variety of risk factors. Smoking is the most important modifiable risk factor for AMD development and progression. The present review summarizes the epidemiological studies evaluating the association between smoking and AMD, the mechanisms through which smoking induces damage to the chorioretinal tissues, and the relevance of advising patients to quit smoking for their visual health.

## 1. Introduction

Age-related macular degeneration (AMD) is a macular neurodegenerative disease that nowadays constitutes one of the main socioeconomical health issues worldwide, afflicting the elderly population. The exponentially increasing prevalence of AMD is linked to progressive aging of the population, and it is the leading cause of legal blindness in the developed world [1–6]. It is traditionally divided into three categories: early AMD, characterized by the presence of pigmentary changes of the retinal pigment epithelium (RPE) and/or hard small drusen; intermediate AMD, characterized by the presence of soft large drusen and/or geographic atrophy (GA) of the RPE with foveal sparing; and late AMD, characterized by GA with foveal involvement and/or choroidal neovascularization (CNV) [7, 8].

On the other hand, the presence or absence of CNV makes the distinction between neovascular AMD (presence of macular fluid and/or hemorrhage secondary to CNV) and atrophic or nonneovascular AMD (presence of any other AMD sign except for CNV). Neovascular AMD accounts for the most cases of severe vision loss, although the atrophic form is the most frequent presentation of the disease [7–10].

A variety of risk factors for AMD have been described. However, the evidence and strength of such associations are widely variable, probably due to the difficulty of measuring some of these factors in clinical practice [11, 12]. Advanced age, Caucasian race, certain genetic polymorphisms, higher body mass index, excessive alcohol consumption, and a history of smoking are proven risk factors in the development of AMD and progression to late AMD [13–19].

Risk factors may be classified as modifiable and nonmodifiable (Table 1). They can also be divided depending on the grade of evidence showed in the literature. Age showed the highest evidence, as the odds ratio (OR) increases from 1 at 55–69 years to 4.42–8.70 at 70–79 years and 18.8–32.3 in ages between 80 and 86 years [20–22]. Smoking (OR range: 2.39–4.22) is the second most consistent risk factor related with AMD [11, 23]. Race and ethnicity may also play an important role, as the whites are the racial group with a higher risk of AMD compared with the blacks or the Hispanic whites [20–22]. Other significant risk factors are family history of AMD (OR range: 3.95–6.98), previous cataract surgery (OR: 1.59), high body mass index (OR range: 1.06–1.35), and hypertension (OR range: 1.02–1.48) [11, 23].

The purpose of this review is to analyze the current scientific evidence of smoking as an independent risk factor in AMD and the relevance of advising patients to quit smoking for their visual health.

## 2. Material and Methods

A systematic review of all of the peer-reviewed articles indexed in PubMed about smoking and age-related macular degeneration was performed. The analyzed data were summarized classifying them into four main headings: reported epidemiological association between smoking and AMD; studied mechanisms for toxic damage to the retina and choroid induced by smoking; smoking and biomarkers in AMD; and treatment considerations for AMD and smoking.

## 3. Results and Discussion

### 3.1. Epidemiological Association between Smoking and AMD

Smoking is a major modifiable risk factor for AMD. The association between smoking and AMD has been consistently demonstrated in many epidemiological studies carried out within different populations in the last decades confirming previous clinical impressions. Cross-sectional studies and prospective cohort studies have described the natural history of the disease and its associations with risk factors, where smoking has been the most consistent factor associated with geographic atrophy and neovascular AMD.

#### 3.1.1. Cross-Sectional Studies

Cross-sectional studies examining the association between smoking and AMD include two American, three European, and two large Australian populations. Further studies also provided additional information about smoking as a risk factor for AMD in different ethnic groups and geographic areas (Table 2). Population-based epidemiologic studies have provided estimates of prevalence and incidence of advanced AMD among various racial/ethnic groups: geographic atrophy and neovascular AMD are rare before 55 years of age, becoming more prevalent in patients aging over 75; overall, the prevalence is higher in Caucasian and lower in African-American patients.

The Beaver Dam Eye Study recruited 4771 patients from Beaver Dam (WI, USA) from 1988. After controlling subjects for age and passive smoking, higher rates of neovascular AMD in current-smokers compared to those who had never smoked independently of gender were evidenced [24].

More recently, the study on the large Beaver Dam Offspring Study (BOSS) cohort found a prevalence of AMD of 3.4%. After controlling subjects for age and gender, a history of current-smoking and greater numbers of pack-years smoked were associated with early AMD [25].

The Rotterdam Study is a single-center prospective study of the population aging over 55 years in Rotterdam (The Netherlands). A total number of 6251 participants were included from 1990 until 1993. Current- and former-smoking was associated with an increased risk of neovascular AMD. This increased risk was present up to 20 years after cessation of smoking. This association was not present in persons aging 85 years and older [26].

The Blue Mountains Eye Study was conducted in Sydney (Australia) between 1992 and 1994. A total number of 3654 patients were included. A highly significant association between current-smokers and geographic atrophy and neovascular AMD was evidenced [27]. Mitchell et al. estimated that 20% of all cases of blindness in Australia may be attributable to smoking, and they advocated a new warning for cigarette packs about the risk of blindness associated with smoking [28]. Later, the Visual Impairment Project (VIP) included 4744 Australian patients and found that total duration of smoking (regardless of the amount) was significantly associated with the prevalence of AMD, suggesting that 14% of all AMD cases might be due to cigarette smoking for longer than 40 years [29].

Pooled data from the aforementioned trials from three different continents provided strong and consistent evidence that tobacco smoking is the main known preventable exposure associated with any form of AMD. Depending on the type of AMD, current-smokers have 2- to 4-fold increase in risk for AMD when compared to patients that never smoked [30].

The Age-Related Eye Disease Study (AREDS) is a longitudinal multicenter study of the natural history of AMD and cataract in 4757 participants recruited between 1992 and 1998. One of the main findings of the AREDS was that smoking was associated with all of the three more severe AMD groups [31].

The EUREYE Study is a cross-sectional study that included 4,750 participants in 2003 from seven centers across Europe. Current-smokers had increased odds of any type of late AMD, and bilateral AMD was also more likely to have a history of heavy smoking. The attributable fraction for AMD due to smoking was 27%.

The Eye Disease Case-Control Study of risk factors for neovascular AMD studied 421 cases and 615 controls between 1986 and 1990, finding a strong association between current cigarette smoking and incidence of AMD [32].

The POLA (Pathologies Oculaires Liées à l'Age) Study included 2196 participants in France. Current- and former-smokers were found to have an increased prevalence of late AMD. This risk was higher in those patients who smoked 20 pack-years and more [33]. The odds ratio (OR) for current-smokers and late AMD observed in this study (3.6) was not significantly different from the OR of 4.46 in the Blue Mountains Eye Study, the OR of 2.2 for neovascular AMD in the Eye Disease Case-Control Study, the OR of 2.38 for late AMD in the VIP Study, the OR of 3.6 for neovascular AMD in the Rotterdam Study, and the OR of 2.5 for women and 3.29 in men for neovascular AMD in the Beaver Dam Eye Study.

Other recent case-control studies show a significant association between both current- and former-smokers and early AMD and polypoidal choroidal vasculopathy in Asian patients [34, 35].

A novel population of monozygotic twins with discordant phenotypes of AMD was studied. Smokers had a twice-fold increased risk of AMD implicating that factors other than DNA sequence are involved in the etiology of AMD.

#### 3.1.2. Prospective Cohort Studies

Prospective cohort study design is most suitable in order to demonstrate that smoking precedes AMD development. There are three key cross-sectional studies that were subsequently extended into longitudinal studies. The length of the follow-up period is the strength of two of them.

At 5-year followup, the Beaver Dam Eye Study reported that men who smoked greater amounts of cigarettes were more likely to develop early AMD. These results were confirmed over a 10-year followup and also evidenced that they were more likely to have progression of the disease [36, 37].

The 5-year incidence of any late AMD lesions found in current, past, or never-smokers in the Blue Mountains Eye Study was 3.1%, 1.2%, and 1.4%, respectively. After adjusting for age, current-smokers had an increased risk of incident geographic atrophy (RR = 3.6; 95% CI: 1.1–11.3) and any late lesions (RR = 2.5; 95% CI: 1.0–6.2) [38]. The long-term incidence over 10 years showed that current-smokers had a 4-fold increase in the risk of late AMD compared with never-smokers (RR = 3.9; 95% CI: 1.7–8.8). Former-smokers had a 3-fold higher risk of geographic atrophy (RR = 3.4; 95% CI: 1.2–9.7) [39]. A further pooled analysis of the 5-year results from these three studies found a continued 3-fold association of current-smoking with the development of AMD [23].

The Physicians' Health Study [40] and the Nurses' Health Study [16] also evidenced that current-smokers of 20 or more cigarettes per day had a 2-fold increased risk compared with never-smokers in two different cohorts of incident cases of AMD followed for at least 7 years.

The AREDS cohort with a median followup of 6.3 years estimated that a larger amount smoked was statistically significantly associated with the incidence of geographic atrophy due to AMD [41]. More recently, Coleman et al. found an increased risk of early AMD among subjects aging 80 years or older who were smokers compared to those younger than 80 years who were not smokers [42].

The risk of smoking has also been estimated in a meta-analysis from six prospective cohort studies, five case-control studies, and five cross-sectional studies. Significant increases in AMD risk were seen for current- versus never-smokers. The odds ratio for case-control studies was 1.78 (95% CI: 1.52–2.09), and it was 3.58 (95% CI: 2.68–4.79) for cross-sectional studies. The relative risk (RR) obtained through analysis of prospective cohort studies was 1.86 (95% CI: 1.27–2.73) [11]. Similar results were also found in a previous meta-analysis [43].

#### 3.1.3. Dose-Response Effect of Smoking

Several studies have investigated the dose-response effect by comparing different levels of smoking classified as pack-years, and most of them confirmed a dose-response effect. The Rotterdam Study and the POLA Study found an increased risk of neovascular AMD in persons who had smoked 10 or more pack-years (OR: 7.1, 95% CI: 2.10–19.0) and 20 or more pack-years (OR: 4.8, 95% CI: 1.80–12.9) [26, 33]. The Physicians' Health Study and the Nurses' Health Study find significant 2-fold higher risk of AMD in persons who had smoked more than 25 cigarettes per day [16, 40]. The VIP Study results reveal that only the duration, not the amount of smoking, is associated with AMD [29]. In the 5-year follow-up results from the Beaver Dam Eye Study the relative risk of early AMD and progression of early AMD increased as the amount smoked increased [36]; the 10-year follow-up results were inconsistent with earlier findings from this and other studies, as no association of the amount smoked or the incidence of late AMD was evidenced [37]. However, the Beaver Dam Offspring Study confirmed that smoking 11 or more pack-years was associated with early AMD [25]. Other studies in Korea and Japan also evidenced that the level of smoking was associated with early and late AMD [34, 44].

#### 3.1.4. The Effect of Quitting Smoking

Exsmokers still have an increased risk of developing AMD compared with never-smokers. The Rotterdam Study was the first to reveal that the increased risk of neovascular AMD remained present up to 20 years after cessation of smoking [26], which was also confirmed in the POLA Study [33]. The Nurses' Health Study reported that past-smoker women who previously smoked 25 or more cigarettes per day had a 2-fold increased risk of AMD even 15 years after cessation of smoking [16]. Other prospective studies found that former-smokers compared with those who never smoked had a modest increased risk of AMD [23].

### 3.2. Mechanisms for Toxic Damage to the Retina and Choroid Induced by Smoking

Cigarette smoke is known to contain an abundant number of toxic compounds. Some of them are known to be either toxic or mutagenic [45]. Its pathological effects through different biochemical pathways and an ocular exposure to cigarette smoke may cause oxidative damage, vascular changes, and inflammation within the pathogenic cascade of AMD. Smoke is responsible for cellular changes at the level of the RPE in AMD patients [46].

#### 3.2.1. Angiogenesis and Neovascularization

Cigarette smoke promotes pathophysiological processes that contribute to atherosclerosis, including thrombosis, vascular inflammation, and endothelial dysregulation [47]. Nicotine itself promotes angiogenesis in experimental models due to its vasculogenic properties [48–50] and may also induce catecholamine release increasing platelet aggregability. Platelets contribute to the growth of plaque through the accretion of thrombus, as well as through the release of growth factors (such as platelet-derived growth factor (PDGF)) that induce vascular smooth muscle cell proliferation. The effect of nicotine also enhances physiological angiogenesis, as observed in wound healing [49, 51] where smoking is known to be a risk factor to delay the wound healing process. In a murine model of CNV, areas that underwent laser-induced rupture of Bruch's membrane are larger in mice after nicotine exposure [51]. Human choroidal and retinal arterial endothelial cells express nicotinic acetylcholine receptors (nAChR), and nicotine enhances their proliferation, migration, and tube-forming ability. Nicotine also exerts a vasoconstrictive action via *α*-adrenergic stimulation which may impair blood flow through the choroid [52].

Nornicotine, a metabolite of nicotine catalyses, can lead to the accumulation of lipofuscin and, therefore, also contribute to the formation of drusen in RPE. Nornicotine can catalyze the alkene isomerization of key retinal intermediates through iminium-ion formation and disrupt proper retinoid homeostasis, revealing an underlying molecular mechanism for tobacco-dependent pathologies, particularly AMD [53].

Cigarette smoke also contains dioxins that are present primarily in the gaseous phase. Most of the toxic effects of dioxins are mediated by the cytosolic dioxin receptor known as aryl hydrocarbon receptor (AhR) [54, 55]. Dioxin acts on ocular tissues through the AhR pathway, promotes vascular endothelial growth factor (VEGF) production in mouse retinal tissues and human RPE cells, and exacerbates the development of laser-induced CNV in mice [56].

#### 3.2.2. Oxidative Damage

The oxidative damage to the RPE contributes to the development and progression of AMD, and the alterations in the metabolic support of the RPE cause apoptosis of the photoreceptors [57, 58]. Cigarette smoke contains a large number of prooxidant compounds. Nicotine promotes nitric oxide (NO) production, and the effect of other proangiogenic growth factors [59]. Cadmium accumulates preferentially in the RPE and choroid [60] and may contribute to the development of AMD through an increase in reactive oxygen species (ROS). However, hydroquinone (HQ) is the most abundant oxidant and is not only in cigarette smoke but also in processed foods, plastic containers, and atmospheric pollutants as well as its widespread occurrence in nature [61, 62].

The RPE cells provide support for the structure and function of the outer retina by secreting several cytokines including monocyte chemoattractant protein-1 (MCP-1) [63, 64]. RPE cells after exposure to HQ can secrete MCP-1 during inflammatory responses promoting macrophage dysfunction. MCP-1 expression is markedly decreased in RPE cells in smoker AMD patients and might play a key role in the pathogenesis of AMD [65, 66]. Both *in vitro* and *in vivo* findings suggest that HQ-induced oxidative damage is unequivocally associated with an imbalance between VEGF and PEDF [65] leading to pathological angiogenesis for the development of CNV [67]. RPE cells from smoker AMD patients exhibit VEGF expression increase and PEDF expression decrease [65–67].

The exposure to cigarette smoke and HQ results in RPE membrane blebbing and sub-RPE deposits in mice. In cultured RPE cells, HQ-induced oxidative injury results in reorganization of actin cytoskeleton and blebs formation important for accumulation of deposits [65–67]. After exposure to HQ, phosphorylate heat shock protein 27 (Hsp27) expression increases, and there is an F-actin reorganization required for RPE-derived bleb formation [65]. Therefore, phosphorylated Hsp27 might be a key mediator in AMD.

Cigarette smoke extract (CSE) is widely used for *in vitro* models [68–70]. CSE causes oxidative damage to human RPE cells *in vitro*, cell death, significantly reduces viability in both ARPE-19 cells and primary RPE cells, via alterations in mitochondrial integrity, and increases lipid peroxidation [71]. Lower concentrations of CSE may induce ROS release and thus cause oxidative stress in primary human RPE cells. Treatment of primary human RPE cultures with CSE could significantly increase the proportion of *β*-galactosidase (SA-*β*-Gal) cells [72]. Sublethal concentrations of hydrogen peroxide have been shown to induce senescence associated to SA-*β*-Gal activity in primary cultured RPE cells [73] and *in vivo* in RPE cells of old primate eyes [74].

Other molecules are involved in oxidative damage. Exposure of primary human RPE cells to CSE may also lead to significant elevations of Apo J, CTGF, and fibronectin expression, which are senescence-associated biomarkers [71]. All three biomarkers are inducible by oxidative stress [75, 76] and have been previously detected in the RPE of AMD donor eyes, although its role and function in the RPE remain unclear.

Oxidative stress is thought to be essential in lipofuscin and drusen formation [77, 78]. Acrolein, an unsaturated aldehyde found in the gas phase of cigarette smoke, exerts an oxidant-mediated damage by inducing protein modifications. RPE cells exposed to acrolein show a decrease in viability and mitochondrial membrane potential due to oxidative stress [79]. In the acrolein RPE model, there is a significant decrease in mitochondrial membrane potential, oxygen consumption, and activity of mitochondrial complexes, and it increased significantly the calcium-ion level [80].

#### 3.2.3. Toxicity

Polycyclic aromatic hydrocarbons (PAHs) are one of the most toxic compounds in cigarette smoke [81]. They form DNA adducts. Benzo(a)pyrene (B(a)P) is a PAH with toxic effects on cultured RPE and RPE/choroid from bovine exposed to chronic cigarette smoke. It causes extensive mitochondrial DNA damage and increases lysosomal activity, formation of a reactive epoxide [82], and caspase-mediated cell apoptosis of human RPE cells [83] perhaps through the generation of epoxides. These altered cell biological processes in the RPE may contribute to the formation of drusen in individuals who are cigarette smokers and underlie susceptibility to genetic mutations associated with AMD.

#### 3.2.4. Experimental Models

ARPE-19 cells, a spontaneously arising human RPE cell line [84], are widely used for *in vitro* studies of cigarette smoke effect in RPE cells. These cells are treated with different toxic substances derived from cigarette smoke such as HQ [85], acrolein [79, 85], CSE [71, 72], B(a)P [86], cadmium [60], and 2,3,7,8-tetrachlorodibenzo-*p*-dioxin (TCDD) [56]. Recently, the effect of the HQ has been studied in a combination of three cellular lines: ARPE-19 cells, rat retinal neurosensory cells (R-28), and human microvascular cells (HMVEC), to demonstrate that nonapoptotic cell death can occur in many forms after the damage [85]. Human donor eyes obtained from the eye bank are processed to obtain human RPE cells. These cellular lines are exposed to HQ [65] and CSE [72] to demonstrate a decrease in the viability after damage. On the other hand, bovine RPE cells also are used in *in vitro* experiments to study the effect of exogenous B(a)P [82]. Other types of experiments include RPE/choroids from mice to analyze the treatment of HQ [56, 65].

#### 3.2.5. *In Vivo* Animal Models

As in *in vitro* models, the same toxic compounds are used in animals to investigate the effect of cigarette smoke in the retina. C57BL/6-pigmented mice are the most widely used in the literature. Hence, these mice receive HQ orally in drinking water for a period of time [65] and are injected intraperitoneally with TCDD [56]. On the other hand, mice are placed into a smoking chamber for a period of time to analyze the effect of the CSE. This chamber contains a smoking machine in different models that burns some cigarettes. Control mice are kept in a filtered-air environment [66, 86, 87]. Cigarette smoke has been also studied in RPE sheets from rats exposed to nicotine in drinking water [65].

#### 3.2.6. Histopathological Changes

The RPE constitutes a cell monolayer that is crucial to maintain a normal photoreceptor function. RPE cell apoptosis and basal deposits, or accumulations of heterogeneous debris in Bruch's membrane, are two critical histopathologic changes that are well recognized to occur during the development of early AMD [88]. Fujihara et al. observed these changes in mice after chronic exposure to cigarette smoke [87], and Espinosa-Heidmann showed that shorter duration and higher concentration of cigarette smoke in old mice induce ultrastructural changes to Bruch's membrane and the choriocapillaris endothelium that are compatible with early AMD [87].

In summary, the most important alterations observed are Bruch's membrane thickening, mild basal deposits and enlargement, and loss of basolateral infoldings, which are an established marker of epithelial cell injury. The formation of vacuoles is a second sign of RPE damage.

## 4. Biomarkers and Smoking in AMD

In order to look for the most appropriate therapies and to individualize lifestyle recommendations, it is ideally necessary to integrate the different clinical features, the habits, and, if available, the biomarkers of a certain disease. A biomarker is a characteristic objectively measured and evaluated as indicator of physiologic/pathologic processes or pharmacologic responses.

Different biomarkers have been studied in AMD patients [89–91]. However, very limited information exists about biomarkers in smokers and type, stage, and progression of AMD or clinical response to treatment. Seddon et al. evaluated the association of serum C-reactive protein (CRP) levels and the risk of AMD, showing that in the smoking population this risk was increased more than 1.7-fold in the lower PCR levels (CRP < 4.5 mg/L). There was no association between smoking and AMD in the highest level of CRP (CRP > 4.5 mg/L). However, the CRP levels were significantly higher among participants with advanced AMD (case patients) than among those with no AMD (controls; median values: 3.4 versus 2.7 mg/L; *P* = .02), so the highest levels of CRP seem to increase the risk of AMD independently of smoking [92].

In a subsequent study, Seddon et al. found that smoking had a positive association with some proinflammatory cardiovascular disease biomarkers such as CRP, interleukin 6, soluble tumor necrosis factor alpha receptor 2, soluble intercellular adhesion molecule-1 (ICAM-1), and apolipoprotein B (ApoB) but not with vascular cell adhesion molecule-1 (VCAM-1) or lipoprotein(a) in nonexudative AMD [93].

Gibson et al. assessed the levels of plasmatic complement component C1 inhibitor (C1inh), and they found that C1inh levels were higher in smokers compared to nonsmokers [94]. These results highlight the importance of considering smoking status in AMD populations.

Other biomarkers have been studied separately in smokers versus nonsmokers and AMD patients versus normal patients and a parallel increase (e.g., increased levels of lipid peroxidation products) and decrease (e.g., decreased levels of antioxidants) of considered markers have been found in both kinds of studies [89, 94–98].

## 5. Treatment Considerations for AMD Smoking Patients

There is limited information about the specific treatment of dry and wet AMD in smokers. The use of antioxidant supplementation consisting of vitamin C (500 mg), vitamin E (400 international units), beta-carotene (15 mg), zinc (80 mg), and copper (2 mg) demonstrated reduction of the risk of progression to advanced dry AMD in the AREDS Study with an average followup of 6.3 years. Some evidence suggested that smokers taking beta-carotene supplementation had an increased risk of lung cancer [99, 100]. However, at the end of the study, the influence of treatment on mortality stratified by smoking status found no effect for current-smokers who took antioxidants. Otherwise, the small proportion of deaths from lung cancer (0.8%) in the AREDS Study showed no difference between treatments [101].

More recently, the AREDS2 Study showed that the addition of omega-3 fatty acids and/or lutein+zeaxanthin to the the original AREDS formulation only reduces by 10% the risk of progression to advanced dry AMD or neovascular AMD [102]. Moreover, there was no effect of beta-carotene elimination or lower zinc dose to the original AREDS formulation on progression to advanced AMD. Given the hypothetic risk of lung cancer due to beta-carotene supplementation, current- or former-smokers within the past year were allowed to participate in the study only in the groups not receiving beta-carotene. The incidence of lung cancer was higher in the beta-carotene (2%) group than in the non-beta-carotene group (0.9%), mainly in former-smokers (91% of participants who developed lung cancer were former-smokers). The simultaneous administration of high doses of beta-carotene and lutein+zeaxanthin may suppress serum and tissue levels of lutein+zeaxanthin due to the competitive absorption of carotenoids. The AREDS2 Study concluded that lutein+zeaxanthin could be a safe carotenoid substitute in the AREDS formulation [102].

In addition, the influence of smoking on the visual outcomes in cases of neovascular AMD treated with intravitreal vascular endothelial growth factor inhibitors has also been analyzed. Lee et al. evidenced that smokers had a statistically significant higher risk (7-fold increase) for a poor response to intravitreal therapies [103], whereas other authors found better functional outcomes in nonsmokers compared with smokers but without statistical significance [104]. The mechanism for the negative influence of cigarette smoking in the response to these therapies is poorly understood.

## 6. Conclusions

Age-related macular degeneration (AMD) is the commonest cause of irreversible visual loss in the Western World [1–6]. AMD is a complex multifactorial disease with an uncertain etiology associated with genetic and environmental risk factors. The hypothesis that interventions trying to minimize the role of such environmental factors may reduce the incidence of AMD and/or the progression to late stages has led to several studies evaluating their relevance in the clinical practice.

Among them, cigarette smoking is a proven risk factor for both development and progression of AMD [24–43], as well as for the clinical response in both atrophic and neovascular forms of AMD [99–104]. As has been previously described, smoking by itself promotes molecular and pathological changes that may establish an ideal macular microenvironment for the development of AMD: vascular inflammation and endothelial dysregulation [47–56], oxidative damage [57–80], toxic damage, and histopathological changes [81–87]. However, patients are not frequently aware of the significant role played by cigarette smoking in blindness associated with AMD. Sometimes, even physicians forget about advising patients of the relevance to quit smoking. Quitting smoking reduces the risk of AMD, and after 20 years of cessation the risk of developing AMD is the same as for nonsmokers [105, 106].

Recently, genetic testing has arisen as an option to provide patients with a certain risk profile based on their own genetic phenotypes in the high-risk genes for AMD [106–108]. This is even more relevant in smoking subjects, as a genetic high-risk profile might influence their motivation to quit smoking [106].

In the situation described above, we believe that institutional support to disseminate the relevance of cigarette smoking in terms of visual health is warranted. Very few countries show health warnings on cigarette packets related to this issue (“SMOKING CAUSES BLINDNESS”), whereas several other health issues warnings are usually included.

## Figures and Tables

**Table 1:** Summary of the nonmodifiable and the modifiable risk factors for age-related macular degeneration*.

Nonmodifiable risk factors
Age
Gender
Iris color
Ethnicity
Hyperopia
Nuclear sclerosis
Family history of AMD
Complement factor H polymorphism
HTRA1 polymorphism
CX3CR1 polymorphism
Complement factors 2, 3 and B polymorphism
Modifiable risk factors
Smoking
Body mass index
Hypertension
Diabetes
Cholesterol
HDL-cholesterol
Acute myocardial infarction
Stroke
Angina
High alcohol intake
Dietary antioxidants and omega-3 fatty acids
Chronic renal failure
Hormone replacement therapy
Chlamydia pneumonie infection
Physical activity
Sunlight exposure
Cataract surgery

*AMD: age-related macular degeneration; HTRA1: HtrA serine peptidase 1; CX3CR1: CX3C chemokine receptor 1; HDL: high-density lipoprotein.

**Table 2:** Cross-sectional and prospective cohort studies examining the association between smoking and AMD: current-smokers versus never-smokers*.

Studies	AMD types	Odds ratio (95% CI)*
Beaver Dam Eye Study	Early AMD Neovascular AMD	Ever-smokers versus never-smokers: M 1.29 (0.98–1.70) F 1.02 (0.81–1.29) Ever-smokers versus never-smokers: M 2.86 (0.64–12.7) F 2.06 (1.03–4.10) Current-smokers versus never-smokers or exsmokers: M 3.29 (1.03–10.50) F 2.50 (1.01–6.20)
Rotterdam Study	Atrophic AMD Neovascular AMD	RR 1.5 (0.6–3.9), RR** 1.9 (0.7–5.4) RR 3.6 (1.8–7.4), RR** 6.6 (2.8–15.9) RR^~^ 0.5 (0.1–4.5)
Blue Mountains Eye Study	Early AMD All late AMD Atrophic AMD Neovascular AMD	1.89 (1.26–2.84) 4.46 (2.20–9.03) 4.94 (1.29–8.82) 3.26 (1.45–7.38)
Visual Impairment Project	AMD	2.38 (0.83–6.80), *P* = 0.11
Beaver Dam Offspring Study	Early AMD	1.83 (1.06–3.17)
Age-Related Eye Disease Study (AREDS)	Early AMD Atrophic AMD Neovascular AMD	Ever-smokers versus never-smokers: 1.25 (1.09–1.44), *P* < 0.01 1.61 (1.06–2.42), *P* < 0.05 1.91 (1.57–2.33), *P* < 0.01
EUREYE Study	Atrophic AMD Neovascular AMD	Smoking in previous 25 years 2.27 (1.27–4.05) 2.86 (1.69–4.85)
Eye Disease Case-Control Study Group	Neovascular AMD	2.20 (1.40–3.50), *P* = 0.002
POLA Study	Late AMD	3.6 (1.0–12.5)
Moon BG, 2012	Early AMD	1.53 (1.37–1.94), *P* = 0.02
Cacket P, 2011	PCV Neovascular AMD	2.8 (1.4–5.5), *P* = 0.002 4.3 (2.1–8.5), *P* < 0.001

Prospective Cohort Study	AMD types	Relative risk (95% CI)*

Physicians' Health Study	Neovascular AMD	2.10 (1.01–4.37), *P* < 0.001
Nurses' Health Study	All AMD	Current smokers 1.70 (1.20-2.50), Women who smoked 25 or more cigarettes per day 2.4 (1.4-4.0), *P* < 0.04
Beaver Dam Eye Study, 5 years	Early AMD Progression of AMD	M 1.53 (0.81–2.99) F 0.74 (0.40–1.35) M 2.34 (1.00–5.44) F 1.00 (0.50–2.01)
Beaver Dam Eye Study, 10 years	Early AMD Late AMD Progression of AMD	1.37 (0.98–1.94) 0.51 (0.18–1.46) 1.34 (0.94–1.91)
Blue Mountains Eye Study, 5 years	Geographic atrophy Neovascular AMD Any late AMD	3.6 (1.1–11.3) 1.6 (0.4–3.7) 2.5 (1.0–6.2)
Blue Mountains Eye Study, 10 years	Geographic atrophy Neovascular AMD Any late AMD	10.3 (2.7–39.1) 1.9 (0.6–5.3) 3.9 (1.7–8.8)
AREDS followup	Geographic atrophy	1.82 (1.25–2.65) 1.55 (1.15–2.09)
Coleman AL, 2010	Early AMD Late AMD	OR 1.11 (0.53–1.23) OR 1.04 (0.31–3.47)

*AMD: age-related macular degeneration; *age and sex adjusted; **less than 85 years; ^~^more than 85 years; CI: confidence interval; M: male; F: female; RR: relative risk; POLA: Pathologies Oculaires Liées à l'Age; PCV: polypoidal choroidal vasculopathy; OR: odds ratio.

## References

[1] Attebo K, Mitchell P, Smith W (1996). Visual acuity and the causes of visual loss in Australia: the Blue Mountains Eye Study. *Ophthalmology*.

[2] Maguire MG, Bressler SB, Bresskr NM (1997). Risk factors for choroidal neovascularization in the second eye of patients with juxtafoveal or subfoveal choroidal neovascularization secondary to age-related macular degeneration. *Archives of Ophthalmology*.

[3] Klaver CCW, Wolfs RCW, Vingerling JR, Hofman A, De Jong PTVM (1998). Age-specific prevalence and causes of blindness and visual impairment in an older population: the Rotterdam study. *Archives of Ophthalmology*.

[4] Casten RJ, Rovner BW, Tasman W (2004). Age-related macular degeneration and depression: a review of recent research. *Current Opinion in Ophthalmology*.

[5] Friedman DS, O’Colmain BJ, Muñoz B (2004). Prevalence of age-related macular degeneration in the United States. *Archives of Ophthalmology*.

[6] Lotery A, Xu X, Zlatava G, Loftus J (2007). Burden of illness, visual impairment and health resource utilisation of patients with neovascular age-related macular degeneration: results from the UK cohort of a five-country cross-sectional study. *British Journal of Ophthalmology*.

[7] Jager RD, Mieler WF, Miller JW (2006). Age-related macular degeneration. *New England Journal of Medicine*.

[8] Gallego-Pinazo R, Dolz-Marco R, Díaz-Llopis M (2012). Towards the new spectral-domain optical coherence tomography based classification of age-related macular degeneration. *Archivos de la Sociedad Española de Oftalmología*.

[9] Klein R, Klein BEK, Jensen SC, Meuer SM (1997). The five-year incidence and progression of age-related maculopathy: the beaver dam eye study. *Ophthalmology*.

[10] Seddon JM, Schachat AP, Ryan S (2001). Epidemiology of age-related macular degeneration. *Retina*.

[11] Chakravarthy U, Wong TY, Fletcher A (2010). Clinical risk factors for age-related macular degeneration: a systematic review and meta-analysis. *BMC Ophthalmology*.

[12] Klein R, Peto T, Bird A, Vannewkirk MR (2004). The epidemiology of age-related macular degeneration. *American Journal of Ophthalmology*.

[13] Seddon JM, Sobrin L, Albert D, Miller J, Azar D, Blodi B (2008). Epidemiology of age-related macular degeneration. *Albert and Jakobiec’s Principles and Practice of Ophthalmology*.

[14] de Jong PTVM (2006). Age-related macular degeneration. *New England Journal of Medicine*.

[15] Seddon JM, Ajani UA, Sperduto RD (1994). Dietary carotenoids, vitamins A, C, and E, and advanced age-related macular degeneration. *Journal of the American Medical Association*.

[16] Seddon JM, Willett WC, Speizer FE, Hankinson SE (1996). A prospective study of cigarette smoking and age-related macular degeneration in women. *Journal of the American Medical Association*.

[17] Seddon JM, Cote J, Davis N, Rosner B (2003). Progression of age-related macular degeneration: association with body mass index, waist circumference, and waist-hip ratio. *Archives of Ophthalmology*.

[18] Seddon JM, Cote J, Rosner B (2003). Progression of age-related macular degeneration: association with dietary fat, transunsaturated fat, nuts, and fish intake. *Archives of Ophthalmology*.

[19] Seddon JM, Rosner B, Sperduto RD (2001). Dietary fat and risk for advanced age-related macular degeneration. *Archives of Ophthalmology*.

[20] Klein R, Klein BEK, Linton KLP (1992). Prevalence of age-related maculopathy. The Beaver Dam Eye Study. *Ophthalmology*.

[21] Vingerling JR, Dielemans I, Hofman A (1995). The prevalence of age-related maculopathy in the Rotterdam study. *Ophthalmology*.

[22] Mitchell P, Smith W, Attebo K, Jie Jin Wang JJW (1995). Prevalence of age-related maculopathy in Australia: the blue mountains eye study. *Ophthalmology*.

[23] Tomany SC, Wang JJ, Van Leeuwen R (2004). Risk factors for incident age-related macular degeneration: pooled findings from 3 continents. *Ophthalmology*.

[24] Klein R, Klein BEK, Linton KLP, DeMets DL (1993). The Beaver Dam Eye Study: the relation of age-related maculopathy to smoking. *American Journal of Epidemiology*.

[25] Klein R, Cruickshanks KJ, Nash SD (2010). The prevalence of age-related macular degeneration and associated risk factors. *Archives of Ophthalmology*.

[26] Vingerling JR, Hofman A, Grobbee DE, De Jong PTVM (1996). Age-related macular degeneration and smoking: the Rotterdam study. *Archives of Ophthalmology*.

[27] Smith W, Mitchell P, Leeder SR (1996). Smoking and age-related maculopathy: the blue mountains eye study. *Archives of Ophthalmology*.

[28] Mitchell P, Chapman S, Smith W (1999). Smoking is a major cause of blindness. *Medical Journal of Australia*.

[29] McCarty CA, Mukesh BN, Fu CL, Mitchell P, Jie Jin Wang JJW, Taylor HR (2001). Risk factors for age-related maculopathy: the visual impairment project. *Archives of Ophthalmology*.

[30] Smith W, Assink J, Klein R (2001). Risk factors for age-related macular degeneration: pooled findings from three continents. *Ophthalmology*.

[31] Ardourel JE (2000). Risk factors associated with age-related macular degeneration: a case-control study in the Age-Related Eye Disease Study: age-related eye disease Study report number 3. *Ophthalmology*.

[32] (1992). Risk factors for neovascular age-related macular degeneration. The Eye Disease Case-Control Study Group. *Archives of Ophthalmology*.

[33] Delcourt C, Diaz J-L, Ponton-Sanchez A, Papoz L (1998). Smoking and age-related macular degeneration: the POLA study. *Archives of Ophthalmology*.

[34] Moon BG, Joe SG, Hwang JU, Kim HK, Choe J, Yoon YH (2012). Prevalence and risk factors of early-stage age-related macular degeneration in patients examined at a health promotion center in Korea. *Journal of Korean Medical Science*.

[35] Cackett P, Yeo I, Cheung CMG (2011). Relationship of smoking and cardiovascular risk factors with polypoidal choroidal vasculopathy and age-related macular degeneration in Chinese persons. *Ophthalmology*.

[36] Klein R, Klein BEK, Moss SE (1998). Relation of smoking to the incidence of age-related maculopathy. The beaver dam eye study. *American Journal of Epidemiology*.

[37] Klein R, Klein BEK, Tomany SC, Moss SE (2002). Ten-year incidence of age-related maculopathy and smoking and drinking: the Beaver Dam Eye Study. *American Journal of Epidemiology*.

[38] Mitchell P, Jin Wang J, Smith W, Leeder SR (2002). Smoking and the 5-year incidence of age-related maculopathy: the Blue Mountains Eye Study. *Archives of Ophthalmology*.

[39] Tan JSL, Mitchell P, Kifley A, Flood V, Smith W, Jie JW (2007). Smoking and the long-term incidence of age-related macular degeneration: the blue mountains eye study. *Archives of Ophthalmology*.

[40] Christen WG, Glynn RJ, Manson JE, Ajani UA, Buring JE (1996). A prospective study of cigarette smoking and risk of age-related macular degeneration in men. *Journal of the American Medical Association*.

[41] Milton RC, Clemons TE, Klien R, Seddon JM, Ferris FL (2005). Risk factors for the incidence of advanced age-related macular degeneration in the Age-Related Eye Disease Study (AREDS): AREDS report no. 19. *Ophthalmology*.

[42] Coleman AL, Seitzman RL, Cummings SR (2010). The association of smoking and alcohol use with age-related macular degeneration in the oldest old. The Study of Osteoporotic Fractures. *American Journal of Ophthalmology*.

[43] Cong R, Zhou B, Sun Q, Gu H, Tang N, Wang B (2008). Smoking and the risk of age-related macular degeneration: a meta-analysis. *Annals of Epidemiology*.

[44] Tamakoshi A, Yuzawa M, Matsui M, Uyama M, Fujiwara NK, Ohno Y (1997). Smoking and neovascular form of age related macular degeneration in late middle aged males: findings from a case-control study in Japan. *British Journal of Ophthalmology*.

[45] Fowles J, Dybing E (2003). Application of toxicological risk assessment principles to the chemical constituents of cigarette smoke. *Tobacco Control*.

[46] Roth F, Bindewald A, Holz FG (2004). Keypathophysiologic pathways in age-related macular disease. *Graefe’s Archive for Clinical and Experimental Ophthalmology*.

[47] Benowitz NL (2003). Cigarette smoking and cardiovascular disease: pathophysiology and implications for treatment. *Progress in Cardiovascular Diseases*.

[48] Heeschen C, Jang JJ, Weis M (2001). Nicotine stimulates angiogenesis and promotes tumor growth and atherosclerosis. *Nature Medicine*.

[49] Heeschen C, Chang E, Aicher A, Cooke JP (2006). Endothelial progenitor cells participate in nicotine-mediated angiogenesis. *Journal of the American College of Cardiology*.

[50] Yu M, Liu Q, Sun J, Yi K, Wu L, Tan X (2011). Nicotine improves the functional activity of late endothelial progenitor cells via nicotinic acetylcholine receptors. *Biochemistry and Cell Biology*.

[51] Kiuchi K, Matsuoka M, Wu JC (2008). Mecamylamine suppresses basal and nicotine-stimulated choroidal neovascularization. *Investigative Ophthalmology and Visual Science*.

[52] Zhu B-Q, Parmley WW (1995). Hemodynamic and vascular effects of active and passive smoking. *American Heart Journal*.

[53] Brogan AP, Dickerson TJ, Boldt GE, Janda KD (2005). Altered retinoid homeostasis catalyzed by a nicotine metabolite: implications in macular degeneration and normal development. *Proceedings of the National Academy of Sciences of the United States of America*.

[54] Hoffman EC, Reyes H, Chu F-F (1991). Cloning of a factor required for activity of the Ah (dioxin) receptor. *Science*.

[55] Reyes H, Reisz-Porszasz S, Hankinson O (1992). Identification of the Ah receptor nuclear translocator protein (Arnt) as a component of the DNA binding form of the Ah receptor. *Science*.

[56] Takeuchi A, Takeuchi M, Oikawa K (2009). Effects of dioxin on vascular endothelial growth factor (VEGF) production in the retina associated with choroidal neovascularization. *Investigative Ophthalmology and Visual Science*.

[57] Beatty S, Koh H-H, Phil M, Henson D, Boulton M (2000). The role of oxidative stress in the pathogenesis of age-related macular degeneration. *Survey of Ophthalmology*.

[58] Cai J, Nelson KC, Wu M, Sternberg P. J, Jones DP (2000). Oxidative damage and protection of the RPE. *Progress in Retinal and Eye Research*.

[59] Lee J, Cooke JP (2012). Nicotine and pathological angiogenesis. *Life Sciences*.

[60] Wills NK, Ramanujam VMS, Chang J (2008). Cadmium accumulation in the human retina: effects of age, gender, and cellular toxicity. *Experimental Eye Research*.

[61] Deisinger PJ, Hill TS, English JC (1996). Human exposure to naturally occurring hydroquinone. *Journal of Toxicology and Environmental Health*.

[62] DeCaprio AP (1999). The toxicology of hydroquinone—relevance to occupational and environmental exposure. *Critical Reviews in Toxicology*.

[63] Holtkamp GM, De Vos AF, Peek R, Kijlsta A (1999). Analysis of the secretion pattern of monocyte chemotactic protein-1 (MCP-1) and transforming growth factor-beta 2 (TGF-*β*2) by human retinal pigment epithelial cells. *Clinical and Experimental Immunology*.

[64] Uetama T, Ohno-Matsui K, Nakahama K-I, Morita I, Mochizuki M (2003). Phenotypic change regulates monocyte chemoattractant protein-1 (MCP-1) gene expression in human retinal pigment epithelial cells. *Journal of Cellular Physiology*.

[65] Pons M, Marin-Castaño ME (2011). Cigarette smoke-related hydroquinone dysregulates MCP-1, VEGF and PEDF expression in retinal pigment epithelium in vitro and in vivo. *PLoS ONE*.

[66] Espinosa-Heidmann DG, Suner IJ, Catanuto P, Hernandez EP, Marin-Castano ME, Cousins SW (2006). Cigarette smoke-related oxidants and the development of sub-RPE deposits in an experimental animal model of dry AMD. *Investigative Ophthalmology and Visual Science*.

[67] Bhutto IA, McLeod DS, Hasegawa T (2006). Pigment epithelium-derived factor (PEDF) and vascular endothelial growth factor (VEGF) in aged human choroid and eyes with age-related macular degeneration. *Experimental Eye Research*.

[68] Martey CA, Pollock SJ, Turner CK (2004). Cigarette smoke induces cyclooxygenase-2 and microsomal prostaglandin E2 synthase in human lung fibroblasts: implications for lung inflammation and cancer. *American Journal of Physiology: Lung Cellular and Molecular Physiology*.

[69] Shapiro SD (2004). Smoke gets in your cells. *American Journal of Respiratory Cell and Molecular Biology*.

[70] Baglole CJ, Sime PJ, Phipps RP (2008). Cigarette smoke-induced expression of heme oxygenase-1 in human lung fibroblasts is regulated by intracellular glutathione. *American Journal of Physiology: Lung Cellular and Molecular Physiology*.

[71] Bertram KM, Baglole CJ, Phipps RP, Libby RT (2009). Molecular regulation of cigarette smoke induced-oxidative stress in human retinal pigment epithelial cells: implications for age-related macular degeneration. *American Journal of Physiology: Cell Physiology*.

[72] Yu AL, Birke K, Burger J, Welge-Lussen U (2012). Biological effects of cigarette smoke in cultured human retinal pigment epithelial cells. *PLoS ONE*.

[73] Yu AL, Fuchshofer R, Kook D, Kampik A, Bloemendal H, Welge-Lüssen U (2009). Subtoxic oxidative stress induces senescence in retinal pigment epithelial cells via TGF-*β* release. *Investigative Ophthalmology and Visual Science*.

[74] Mishima K, Handa JT, Aotaki-Keen A, Lutty GA, Morse LS, Hjelmeland LM (1999). Senescence-associated *β*-galactosidase histochemistry for the primate eye. *Investigative Ophthalmology and Visual Science*.

[75] Dumont P, Burton M, Chen QM (2000). Induction of replicative senescence biomarkers by sublethal oxidative stresses in normal human fibroblast. *Free Radical Biology and Medicine*.

[76] Toussaint O, Medrano EE, Von Zglinicki T (2000). Cellular and molecular mechanisms of stress-induced premature senescence (SIPS) of human diploid fibroblasts and melanocytes. *Experimental Gerontology*.

[77] Katz ML, Robison WG (2002). What is lipofuscin? Defining characteristics and differentiation from other autofluorescent lysosomal storage bodies. *Archives of Gerontology and Geriatrics*.

[78] Sparrow JR, Boulton M (2005). RPE lipofuscin and its role in retinal pathobiology. *Experimental Eye Research*.

[79] Jia L, Liu Z, Sun L (2007). Acrolein, a toxicant in cigarette smoke, causes oxidative damage and mitochondrial dysfunction in RPE cells: protection by (R)-*α*-lipoic acid. *Investigative Ophthalmology and Visual Science*.

[80] Liu Z, Sun L, Zhu L (2007). Hydroxytyrosol protects retinal pigment epithelial cells from acrolein-induced oxidative stress and mitochondrial dysfunction. *Journal of Neurochemistry*.

[81] Conney AH (1982). Induction of microsomal enzymes for foreign chemicals and carcinogenesis by polycyclic aromatic hydrocarbons: G.H.A. Clowes Memorial Lecture. *Cancer Research*.

[82] Patton WP, Routledge MN, Jones GD (2002). Retinal pigment epithelial cell DNA is damaged by exposure to benzo[a]pyrene, a constituent of cigarette smoke. *Experimental Eye Research*.

[83] Sharma A, Neekhra A, Gramajo AL (2008). Effects of Benzo(e)Pyrene, a toxic component of cigarette smoke, on human retinal pigment epithelial cells in vitro. *Investigative Ophthalmology and Visual Science*.

[84] Dunn KC, Aotaki-Keen AE, Putkey FR, Hjelmeland LM (1996). ARPE-19, a human retinal pigment epithelial cell line with differentiated properties. *Experimental Eye Research*.

[85] Sharma A, Patil JA, Gramajo AL, Seigel GM, Kuppermann BD, Kenney CM (2012). Effects of hydroquinone on retinal and vascular cells in vitro. *Indian Journal of Ophthalmology*.

[86] Wang L, Clark ME, Crossman DK (2010). Abundant lipid and protein components of drusen. *PLoS ONE*.

[87] Fujihara M, Nagai N, Sussan TE, Biswal S, Handa JT (2008). Chronic cigarette smoke causes oxidative damage and apoptosis to retinal pigmented epithelial cells in mice. *PLoS ONE*.

[88] Spraul CW, Lang GE, Grossniklaus HE (1996). Morphometric analysis of the choroid, Bruch’s membrane, and retinal pigment epithelium in eyes with age-related macular degeneration. *Investigative Ophthalmology and Visual Science*.

[89] Ross RJ, Verma V, Rosenberg KI, Chan CC, Tuo J (2007). Genetic markers and biomarkers for age-related macular degeneration. *Expert Review of Ophthalmology*.

[90] Kim TW, Kang JW, Ahn J Proteomic analysis of the aqueous humor in age-related macular degeneration (AMD) patients. *Journal of Proteome Research*.

[91] Yao J, Liu X, Yang Q (2013). Proteomic analysis of the aqueous humor in patients with wet age-related macular degeneration. *Proteomics*.

[92] Seddon JM, Gensler G, Milton RC, Klein ML, Rifai N (2004). Association between C-reactive protein and age-related macular degeneration. *Journal of the American Medical Association*.

[93] Seddon JM, George S, Rosner B, Rifai N (2005). Progression of age-related macular degeneration: prospective assessment of C-reactive protein, interleukin 6, and other cardiovascular biomarkers. *Archives of Ophthalmology*.

[94] Gibson J, Hakobyan S, Cree AJ (2012). Variation in complement component C1 inhibitor in age-related macular degeneration. *Immunobiology*.

[95] Ozguner F, Koyu A, Cesur G (2005). Active smoking causes oxidative stress and decreases blood melatonin levels. *Toxicology and Industrial Health*.

[96] Jia L, Dong Y, Yang H, Pan X, Fan R, Zhai L (2011). Serum superoxide dismutase and malondialdehyde levels in a group of Chinese patients with age-related macular degeneration. *Aging*.

[97] Shen X, Jia LH, Zhao P (2012). Changes in blood oxidative and antioxidant parameters in a group of Chinese patients with age-related macular degeneration. *Journal of Nutrition, Health and Aging*.

[98] Yanbaeva DG, Dentener MA, Creutzberg EC, Wesseling G, Wouters EFM (2007). Systemic effects of smoking. *Chest*.

[99] Heinonen OP, Albanes D (1994). The effect of vitamin E and beta carotene on the incidence of lung cancer and other cancers in male smokers. *New England Journal of Medicine*.

[100] Omenn GS, Goodman GE, Thornquist MD (1996). Effects of a combination of beta carotene and vitamin A on lung cancer and cardiovascular disease. *New England Journal of Medicine*.

[101] Age-Related Eye Disease Study Research Group (2001). A randomized, placebo-controlled, clinical trial of highdose supplementationwith vitamins C and E, beta carotene, and zinc for age-related macular degeneration and vision loss: AREDS report no. 8. *Archives of Ophthalmology*.

[102] Age-Related Eye Disease Study 2 Research Group (2013). Lutein + zeaxanthin and omega-3 fatty acids for age-related macular degeneration: the Age-Related Eye Disease Study 2 (AREDS2) randomized clinical trial. *Journal of the American Medical Association*.

[103] Lee S, Song SJ, Yu HG (2013). Current smoking is associated with a poor visual acuity improvement after intravitreal ranibizumab therapy in patients with exudative age-related macular degeneration. *Journal of Korean Medical Science*.

[104] McKibbin M, Ali M, Bansal S (2012). CFH, VEGF and HTRA1 promoter genotype may influence the response to intravitreal ranibizumab therapy for neovascular age-related macular degeneration. *British Journal of Ophthalmology*.

[105] Hughes AE, Orr N, Patterson C (2007). Neovascular age-related macular degeneration risk based on CFH, LOC387715/HTRA1, and smoking. *PLoS Medicine*.

[106] Rennie CA, Stinge A, King EA, Sothirachagan S, Osmond C, Lotery AJ (2012). Can genetic risk information for age-related macular degeneration influence motivation to stop smoking A pilot study. *Eye*.

[107] Priya RR, Chew EY, Swaroop A (2012). Genetic studies of age-related macular degeneration: lessons, challenges, and opportunities for disease management. *Ophthalmology*.

[108] Stone EM, Aldave AJ, Drack AV (2012). Recommendations for genetic testing of inherited eye diseases. Report of the American Academy of Ophthalmology Task Force on Genetic Testing. *Ophthalmology*.

